# Macrophage immunometabolism in glioblastoma: from microglial reprogramming to B cell dysfunction

**DOI:** 10.3389/fimmu.2026.1947853

**Published:** 2026-10-26

**Authors:** Elizabeth Yu, Grace Jones, Catalina Lee-Chang

**Affiliations:** 1 Department of Neurological Surgery, Feinberg School of Medicine, Northwestern University, Chicago, IL, United States; 2 Weinberg College of Arts and Sciences, Northwestern University, Evanston, IL, United States; 3 Malnati Brain Tumor Institute of the Lurie Comprehensive Cancer Center, Feinberg School of Medicine, Northwestern University, Chicago, IL, United States

**Keywords:** B cell, glioblastoma, macrophages, microglia, tumor microenvironment, immunometabolism

## Abstract

Glioblastoma (GBM) is an aggressive primary brain tumor whose progression and resistance to therapy are driven not only by malignant cells but also by extensive metabolic and immunological remodeling of the tumor microenvironment (TME). Among the most abundant immune populations within the GBM TME are tumor-associated macrophages (TAMs), including resident microglia and infiltrating monocyte-derived macrophages. Under physiological conditions, microglia preserve central nervous system (CNS) homeostasis through immune surveillance, phagocytosis, synaptic remodeling, and metabolic regulation. However, exposure to tumor-derived signals, hypoxia, and nutrient deprivation reprograms these cells toward predominantly immunosuppressive phenotypes that promote tumor growth, angiogenesis, immune evasion, and therapeutic resistance. Emerging evidence demonstrates that TAMs engage in extensive bidirectional communication with B cells, further shaping the immunosuppressive landscape of GBM. Macrophages regulate B-cell activation, survival, differentiation, and antigen presentation through cytokines, chemokines, growth factors, and direct cell-cell interactions, while B cells influence macrophage polarization through antibody production and inflammatory signaling. Simultaneously, metabolic competition within the TME, characterized by glucose depletion, lactate accumulation, and hypoxia, impairs B-cell effector functions, diminishes antigen presentation and humoral immunity, and promotes the development of regulatory B cells (Bregs), thereby reinforcing immune suppression and facilitating tumor progression. This review summarizes the physiological functions of microglia, the metabolic and functional reprogramming of tumor-associated myeloid populations in GBM, and the emerging evidence surrounding TAM–B-cell interactions. By integrating findings from GBM with mechanistic insights from other tumor and disease contexts, we examine how metabolic constraints within the TME could potentially influence macrophage–B-cell crosstalk. We propose this relationship as a novel and testable framework for understanding immune regulation in GBM, while distinguishing established GBM-specific mechanisms from pathways supported primarily by evidence in other disease settings. Finally, we discuss how macrophage immunometabolism and its potential intersection with B-cell function may represent areas for future investigation and therapeutic exploration in GBM.

## 1 Introduction

GBM is one of the most aggressive primary brain tumors, and is the most common malignant primary brain tumor in adults. In the United States, GBM accounts for approximately 48.6% of all malignant brain and other CNS tumors (1). However, the incidence and proportion of GBM around primary brain tumors varies substantially across countries and populations, with a median survival of approximately 15 months (2, 3). The addition of temozolomide to radiotherapy improved survival in patients with newly diagnosed GBM, establishing an important advanced in standard treatment (4). However, long-term survival remained limited, highlighting the need to better understand the biological mechanisms that contribute to tumor persistence and resistance to therapy (5).

One of the primary factors underlying the resilience of GBM is its TME. This is notably characterized by a complex and heterogeneous network of malignant astrocytic cells, stromal components, blood vessels, immune cells, and necrotic regions embedded within a highly dynamic extracellular matrix (6). The TME is highly immunosuppressive, hindering anti-tumor immune responses and enabling tumor persistence by preventing the immune system from combatting tumors (7). The cellular and metabolic conditions within this environment can alter the behavior of immune cells and contribute to the persistence of immune suppression (8).

The TME consists of several critical immune cell populations that contribute to GBM progression. TAMs, which include both resident microglia and infiltrating monocytes, make up to 30% of the tumor mass and secrete cytokines that suppress the immune response and promote tumor proliferation, invasion, angiogenesis, and immune suppression (9). Myeloid-derived suppressor cells (MDSCs) further contribute to this immunosuppressive environment by inhibiting T cell function and facilitating persistence (7). Among these populations, macrophages are particularly relevant to the metabolic regulation of the GBM immune microenvironment.

Macrophages are specialized tissue-resident innate immune cells that play essential roles in maintaining homeostasis, coordinating inflammatory responses, and defending against pathogens. Although macrophages are often categorized as M1 or M2, their activation state is more like a spectrum. In tumors, macrophages are frequently skewed toward phenotypes that suppress immune responses and alter communication with both T and B cells (10). In the CNS, microglia make up about 10% of all CNS cells and exhibit specific responses based on the different brain regions. Unlike peripheral macrophages, it is believed that microglia originate from embryonic yolk sac (YS) progenitors and maintain their population through local self-renewal (11, 12). Under physiological conditions, microglia contribute to CNS homeostasis through immune monitoring, clearance of cellular debris, and regulation of neuronal development and function (12, 13).

During brain tumor development, resident microglia, along with infiltrating bone marrow-derived myeloid cells, undergo metabolic and functional reprogramming in response to tumor-derived signals, hypoxia, and nutrient competition within the TME (14). These tumor-associated changes extend beyond alterations in immune cell abundance and phenotype. Increasing evidence suggests that metabolic reprogramming is a central driver of microglia and myeloid cell dysfunction within GBM. Microglia rely mainly on oxidative metabolism to support immune surveillance, tissue maintenance, and neuronal homeostasis. However, tumor-associated metabolic stressors can induce profound shifts in cellular metabolism that disrupt inflammatory signaling and impair antigen presentation, phagocytic capacity, and crosstalk with other immune populations. As a result, microglia frequently transition from homeostatic regulators of the CNS to active participants in the immunosuppressive TME that supports GBM progression and therapeutic resistance (15). These changes raise the broader question of how metabolically reprogrammed macrophages influence other immune populations within the TME.

In addition to influencing myeloid cell behavior, the metabolic and immunological landscape of GBM shapes communication between microglia, TAMs, and adaptive immune cells, including B lymphocytes. Although B cells have traditionally been studied for their antibody-producing functions, emerging evidence demonstrates that they also participate in antigen presentation, cytokine production, and regulation of innate immune responses, allowing them to influence the activity and polarization of macrophages and other myeloid populations. Emerging evidence suggests that bidirectional interactions between B cells and macrophages play an important role in regulating local immune responses, with B cell derived factors promoting anti-inflammatory macrophage phenotypes and shaping tissue inflammatory states (16). However, B cells constitute a relatively small proportion of the immune compartment in GBM, and the functional significance of macrophage—B cell interactions in this setting are incompletely understood.

Importantly, the extent to which these interactions contribute to GBM biology remains an open question. While macrophage–B-cell crosstalk has been described in other tumor and disease contexts, direct evidence defining these interactions in GBM remains limited. Nevertheless, the profound metabolic reprogramming experienced by macrophages within the GBM TME raises the possibility that alterations in macrophage metabolism could influence B-cell recruitment, activation, differentiation, or function. Conversely, B-cell-derived signals may further shape macrophage states within the metabolically constrained GBM TME. We therefore propose, as a hypothetical and testable framework, that metabolic reprogramming of tissue-resident and infiltrating macrophages may represent an underappreciated determinant of B-cell function in GBM. This framework is intended to generate hypotheses for future investigation rather than to imply that macrophage–B-cell metabolic crosstalk has been established as a mechanism in GBM. This review therefore examines how metabolic reprogramming of tissue-resident macrophages may shape B-cell immunity during GBM progression, with particular emphasis on potential mechanisms underlying macrophage–B-cell crosstalk. By integrating evidence directly demonstrated in GBM with mechanistic insights from other tumor and disease contexts, we highlight potential pathways through which macrophage immunometabolism could influence B-cell function and identify key questions that require validation in GBM. Ultimately, this emerging framework may provide a basis for investigating whether macrophage–B-cell interactions represent a therapeutically relevant component of the GBM immune microenvironment.

## 2 Microglial biology under physiological conditions

### 2.1 Central nervous system resident macrophages: origin and maintenance

The CNS contains specialized populations of tissue-resident macrophages that play essential roles in immune surveillance, tissue homeostasis, and responses to injury and disease. These macrophages can be broadly classified into two major populations: parenchymal microglia and border-associated macrophages (BAMs) (11). BAMs occupy CNS border regions, including the meninges, perivascular spaces, and choroid plexus, and arise from both YS- and fetal liver-derived progenitors (17).

Microglia are recognized as a distinct CNS-resident myeloid lineage that originates from primitive YS derived macrophages that colonize the developing brain before formation of the blood-brain barrier (BBB) (12). Recent fate-mapping studies in mice demonstrated that microglia are embryonically derived from YS macrophages and persist throughout life through local self-renewal rather than continuous replenishment by BM derived monocytes. Once they establish themselves in the brain, they are long-lived, persisting for months to years (13, 18). Similar developmental patterns have also been described for BAMs, which are largely established during embryogenesis and maintained through self-renewal during steady-state conditions (19).

This distinction is particularly relevant in GBM, where the tumor-associated myeloid compartment includes both resident microglia and monocyte-derived macrophages recruited from the circulation. In GBM, these populations exhibit distinct spatial distributions, with infiltrating macrophages preferentially localized to perivascular regions and resident microglia mainly localized to peritumoral regions (20).

Microglia are highly adaptable cells that can alter their morphology and function in response to environmental cues (21). This plasticity enables microglia to efficiently remove microbes, apoptotic cells, protein aggregates, redundant synapses, and other potentially harmful antigens that threaten CNS integrity (22).

### 2.2 Microglial homeostasis and metabolism

Microglia are central regulators of CNS homeostasis, a highly regulated state that depends on coordinated interactions among neurons, glial cells, and BBB to preserve ionic equilibrium, metabolic support, and immune regulation. As the resident innate immune cells of the CNS, microglia continuously survey their environment for both external threats, such as invading pathogens, and internal danger signals from damaged or dying cells. Detection of these signals initiates protective responses that promote tissue repair and debris clearance while limiting excessive inflammatory damage (13).

Under physiological conditions, microglia maintain a relatively downregulated phenotype compared with other tissue macrophages, characterized by low expression of immune receptors but enrichment of genes related to oxidative metabolism and cellular maintenance functions. Neurons and astrocytes actively contribute to this homeostatic state through inhibitory signaling pathways that suppress excessive microglial activation while preserving the capacity for rapid responses to injury or infection (23). When neurons become damaged or undergo degeneration, this inhibitory control can be removed, allowing microglia to adopt more reactive phenotypes that contribute to inflammatory responses and disease progression (23).

The ability of microglia to maintain CNS homeostasis is closely linked to their unique metabolic profile. In healthy CNS, microglia primarily rely on mitochondrial oxidative phosphorylation (OXPHOS) for ATP production, utilizing glucose through oxidative metabolism rather than glycolysis (24, 25). Although resting microglia exhibit relatively low glycolytic activity, they retain considerable metabolic flexibility and can rapidly shift toward glycolysis during inflammation or tissue injury to support increased energy demands and immune activation. This metabolic plasticity also allows microglia to utilize alternative energy substrates and adjust their metabolic activity in response to environmental changes (24).

In addition to glucose metabolism, lipid and cholesterol metabolism play critical roles in maintaining microglial function. Beyond serving as energy substrates, lipids are integral to membrane structure, signal transduction, and broader cell regulations. Homeostatic microglia express a transcriptional profile that supports lipid uptake, transport, and metabolism, sustaining their maintenance and surveillance functions (19).

Maintenance of oxidative metabolism and low glycolytic activity is closely associated with the homeostatic microglial phenotype. Excessive glycolytic activity and mitochondrial dysfunction can disrupt this balance by promoting the production of reactive oxygen species (ROS) and pro-inflammatory mediators (26).

### 2.3 Microglial functions in CNS development and maintenance

The homeostatic and metabolic properties of microglia enable them to perform a wide range of functions that are essential for normal CNS development and maintenance. During prenatal development, microglia are the first glial cells to enter the CNS, where they localize neuronal migratory routes and axonal pathways to facilitate the establishment of complex neural networks (27). Microglia also contribute to neurogenesis and tissue maintenance by responding to local signals and removing apoptotic cells through phagocytosis (22, 28). During postnatal development, complement-tagged synapses that fail to establish functional connections are removed through phagocytosis to shape mature neural networks (29).

## 3 Microglia and myeloid reprogramming in GBM

TAMs, consisting of both resident microglia and infiltrating monocyte-derived macrophages, represent the majority of the myeloid population within the GBM TME (30). These cells play central roles in tumor initiation and progression. Although the traditional M1/M2 polarized model is recognized as an oversimplification of myeloid cell classifications, GBM-associated TAMsexhibit diverse functional states that can promote tumor growth and suppress antitumor immune responses (31).

While both resident microglia and infiltrating macrophages contribute to glioma progression, accumulated evidence suggests that they occupy distinct spatial niches and exhibit complementary functions within the TME. Microglia are mainly enriched at the invasive tumor margin, where they facilitate tumor migration and recruit additional immunosuppressive immune populations (32, 33). In contrast, infiltrating macrophages are more commonly localized to hypoxic tumor regions and are closely associated with angiogenesis, vascular remodeling, and tumor recurrence. In addition to TAMs, myeloid derived suppressor cells (MDSCs) comprise another immunosuppressive myeloid population within GBM. These immature myeloid cells suppress T cell activation and proliferation through multiple mechanisms, limiting effective antitumor immunity, and further promoting tumor progression (7).

### 3.1 Metabolic reprogramming in the GBM microenvironment

Metabolic reprogramming is a hallmark of GBM that enables sustained tumor proliferation and promotes immune evasion. To meet the high demands of rapid growth, GBM cells undergo metabolic adaptations that reshape the surrounding TME into a hypoxic and nutrient-deprived niche that favors immunosuppressive immune populations over effective antitumor responses (14). As tumor cells proliferate rapidly, their growth outpaces the developing vasculature, resulting in regions of oxygen deprivation and limited nutrient availability (34). Because of this, GBM cells increasingly rely on glycolysis rather than OXPHOS, generating metabolic byproducts that further reinforce the immunosuppressive microenvironment while stimulating angiogenesis to restore nutrient and oxygen supply and sustain continued tumor growth (35, 36).

These metabolic alterations also influence the composition and function of tumor-associated myeloid populations. TAM composition differs between primary and metastatic brain tumors, reflecting the dynamic nature of the GBM microenvironment (31). Single-cell transcriptomic studies have identified multiple TAM subpopulations, including transient-state, glia-like, hypoxic, and phagocytic lipid-rich macrophages, each exhibiting distinct transcriptional profiles and functional properties (31). This heterogeneity is strongly influenced by local microenvironmental conditions, including oxygen availability, metabolic stress, and disease state, resulting in differences between primary and recurrent tumors that further contribute to immune complexity within GBM (31).

### 3.2 Functional reprogramming of microglia and macrophages

Microglia exhibit functional plasticity and can transition between activation states in response to changes in their surrounding microenvironment. Although the classical M1/M2 polarization has historically been used to describe pro-inflammatory/immune-activating M1 and anti-inflammatory/tissue repair M2 responses, it does not fully capture the complexity of microglia activation *in vivo* (37, 38). Glioma-associated microglia/macrophages have been shown to exhibit expression profiles distinct from canonical M1 and M2 states, with individual cells displaying features of both phenotypes (39).

Rather than transitioning between discrete M1 and M2 states, microglia can adopt diverse functional states shaped by local environmental cues, including inflammatory signals, nutrient availability, metabolites, and other features of the TME (40). In GBM, tumor-derived signals can induce functional reprogramming of microglia, contributing to their tumor-supportive activities (41) This reprogramming is also accompanied by metabolic adaptations involving pathways such as glucose and lipid metabolism and responses to hypoxic conditions (42, 43). Recent studies have identified distinct myeloid states associated with features such as hypoxia, lipid metabolism, phagocytic activity, and interferon responses, highlighting the transcriptional and metabolic heterogeneity of myeloid cells within the GBM microenvironment (40, 43).

Microglial functional states are also accompanied by changes in cellular morphology. In the healthy CNS, microglia exhibit a highly ramified morphology with processes that continuously survey the surrounding environment. Following disruption of CNS homeostasis, microglia undergo morphological and transcriptional changes and may adopt a more amoeboid phenotype associated with activated immune functions (12, 44). These morphological changes occur alongside broader transcriptional and metabolic adaptations rather than representing a uniform transition between M1 and M2 states (40, 43). However, chronic activation can become detrimental, contributing to persistent neuroinflammation associated with neurodegenerative and neoplastic diseases. Within GBM, tumor-derived signals exploit this inherent plasticity by driving microglia toward tumor supportive phenotypes that suppress antitumor immunity while also producing growth factors that promote tumor survival (44).

### 3.3 Myeloid heterogeneity in GBM

Microglia and monocyte-derived macrophages are distinct myeloid populations that contribute to the brain TME. Microglia are long-lived, self-renewing resident immune cells of the CNS parenchyma that originate from embryonic YS progenitors, whereas monocyte-derived macrophages arise from circulating bone marrow-derived monocytes that infiltrate the CNS under inflammatory and pathological conditions (16, 45). In the context of brain tumors, both populations can be reprogrammed toward tumor-supportive phenotypes (45).

In GBM and brain metastases, both resident microglia and infiltrating macrophages accumulate within and around tumor masses and are commonly grouped as tumor-associated microglia/macrophages (TAMs) (16). Because these populations share numerous myeloid markers, including CD11b and Iba1, distinguishing them can be challenging. However, microglia are generally characterized as CD11b^+^CD45^low cells, whereas infiltrating monocyte-derived macrophages exhibit a CD11b^+^CD45^high phenotype (46, 47). Microglia are frequently observed at the tumor margin and surrounding parenchyma, whereas infiltrating macrophages are often enriched within the tumor core and regions of BBB disruption (16). Collectively, these tumor-associated myeloid populations can comprise up to 30-50% of the total GBM tumor mass, underscoring their central role in shaping the TME and influencing disease progression (16).

Both microglia and monocyte-derived macrophages contribute to an immunosuppressive TME by secreting cytokines, growth factors, and matrix-remodeling enzymes that promote tumor proliferation, invasion, angiogenesis, and immune evasion. Following tumor-mediated reprogramming, these cells often adopt tumor-supportive phenotypes that facilitate GBM progression rather than antitumor immune responses (9, 16).

Despite their shared markers and overlapping tumor-supportive functions, resident microglia and monocyte-derived macrophages occupy distinct metabolic and spatial niches. Single-cell and spatial analyses indicate that microglia-derived TAMs, which predominate at the invasive margin, retain a comparatively oxidative, lipid-handling metabolic program and express pro-inflammatory and interferon-associated signatures, whereas monocyte-derived TAMs accumulate in the hypoxic, perivascular, and necrotic tumor core, adopt a more glycolytic profile, and preferentially upregulate immunosuppressive mediators (48, 49). The two compartments behave as self-renewing populations that compete for tumor territory, with microglia-derived TAMs predominating in newly diagnosed tumors and monocyte-derived TAMs expanding at recurrence, particularly within hypoxic regions (49). These ontogeny-linked metabolic differences carry therapeutic weight, since strategies that indiscriminately target all TAMs may spare the more immunosuppressive, blood-derived population most strongly associated with poor outcomes (48).

GBM-derived chemokines and inflammatory mediators recruit circulating monocytes into the CNS, where they differentiate into macrophages and further support tumor progression (16).

MDSCs represent a third immunosuppressive myeloid population that accumulates in GBM and other CNS malignancies. Unlike microglia and differentiated macrophages, MDSCs are immature myeloid cells that expand during chronic inflammation and cancer. These cells suppress antitumor immunity through inhibition of T-cell activation, promotion of T-cell exhaustion, production of immunosuppressive cytokines, and support of tumor-associated immune tolerance (16).

Therefore, although microglia, monocyte-derived macrophages, and MDSCs are frequently grouped together within the tumor-associated myeloid compartment, they differ in developmental origin, localization, and functional specialization. Microglia are embryonically derived CNS-resident immune cells, monocyte-derived macrophages originate from bone marrow-derived circulating monocytes, and MDSCs are immature myeloid cells with potent immunosuppressive activity. Distinguishing these populations is vital for understanding GBM-associated immune suppression, as depicted in **Figure 1**, and for developing therapies that selectively target pro-tumor myeloid populations while preserving beneficial immune functions.

**Figure 1:**
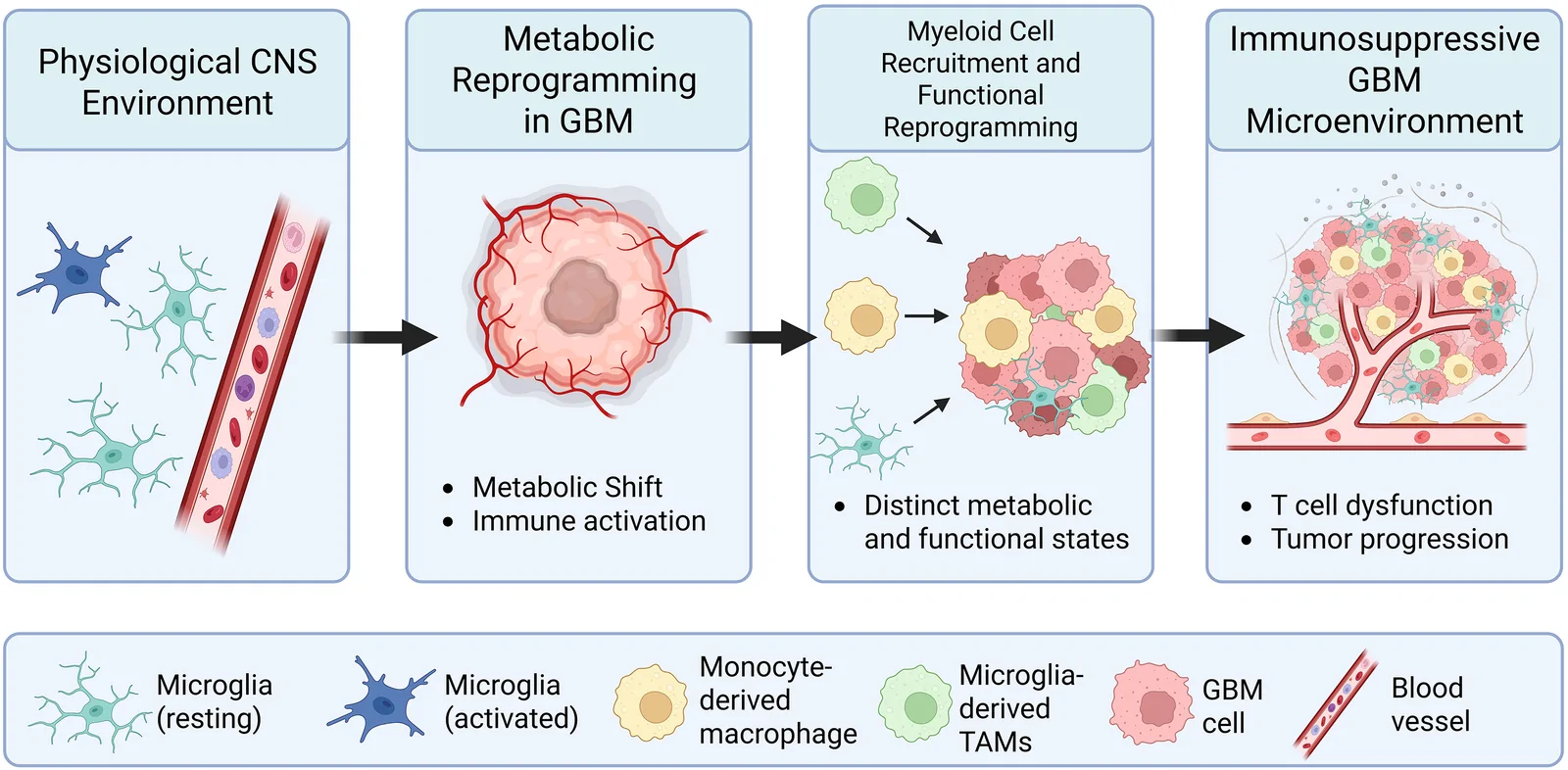
Overview of microglial metabolic reprogramming and myeloid remodeling in glioblastoma. Homeostatic microglia maintain CNS immune surveillance and tissue homeostasis under physiological conditions. During GBM progression, tumor-derived signals induce metabolic reprogramming of resident microglia and infiltrating monocyte-derived macrophages, promoting their polarization into immunosuppressive TAMs. Recruitment of additional myeloid populations, including MDSCs, further amplifies immune suppression, resulting in T-cell dysfunction, angiogenesis, and tumor progression. These metabolically reprogrammed myeloid populations also establish the immunological landscape that facilitates subsequent crosstalk with adaptive immune cells, including B cells, discussed throughout this review.

B cells and tumor-associated macrophages (TAMs) engage in bidirectional interactions that contribute to the regulation of immune responses within the tumor microenvironment (TME), as illustrated in **Figure 2**. B cells can influence macrophage polarization states, shaping inflammatory or immunosuppressive outcomes depending on the context. In turn, macrophages support B cell biology through the secretion of soluble mediators and antigen-presenting signals. These signals promote B cell proliferation and then differentiation into plasmablasts, plasma cells, and germinal center B cells. Together, these interactions show the relationship between B cells and myeloid populations in regulating immune responses (50, 51).

**Figure 2:**
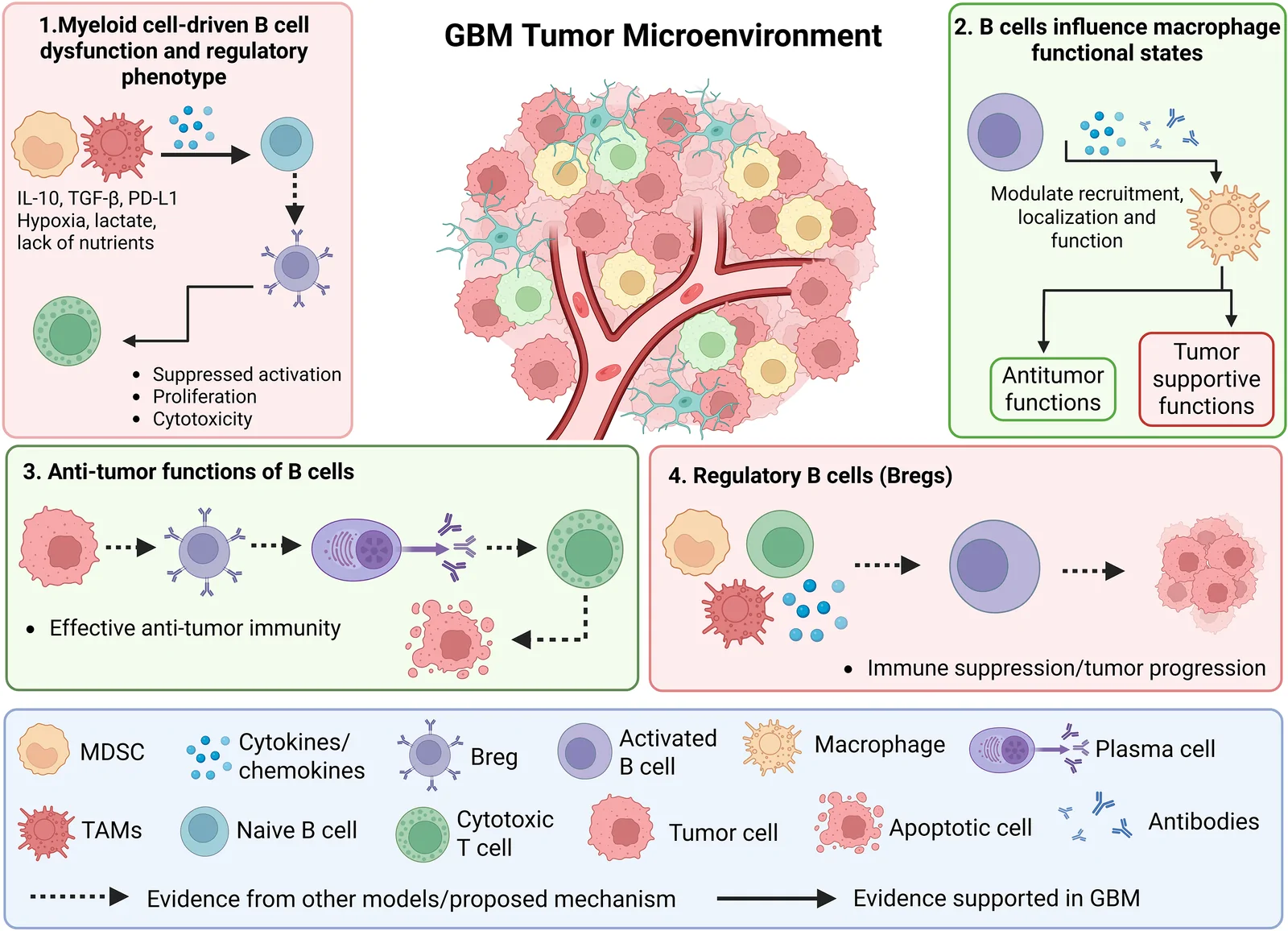
Bidirectional crosstalk between TAMs and B cells in the glioblastoma microenvironment. The GBM TME promotes reciprocal interactions between TAMs and B cells that collectively reinforce immune suppression and tumor progression. (1) Tumor-derived factors and immunosuppressive TAMs generate a metabolically hostile microenvironment characterized by hypoxia, nutrient depletion, lactate accumulation, and immunosuppressive cytokines (e.g., IL-10, TGF-β, and PD-L1), impairing B-cell activation, proliferation, cytotoxicity, and antigen presentation while promoting the differentiation of regulatory B cells (Bregs). (2) Activated B cells reciprocally regulate macrophage functional states through cytokine secretion, promoting either pro-inflammatory M1 macrophages that support antitumor immunity or immunosuppressive M2 macrophages that facilitate tumor growth and immune evasion. (3) Under favorable conditions, activated B cells differentiate into plasma cells that produce tumor-specific antibodies, enhancing antibody-dependent cellular cytotoxicity, macrophage-mediated phagocytosis, and adaptive antitumor immune responses. (4) Conversely, Bregs suppress cytotoxic T-cell function and reinforce the immunosuppressive GBM microenvironment, creating a positive feedback loop that supports tumor progression.

An important caveat frames this section. GBM is an immunologically “cold” tumor in which lymphocyte infiltration is sparse, and the immune compartment is dominated by myeloid cells; B cells in particular constitute only a minor fraction of the tumor-infiltrating population (52). Much of the macrophage–B cell crosstalk discussed below is therefore inferred from peripheral lymphoid tissue or from other, more B-cell-rich tumor types, and its magnitude and functional significance within the GBM microenvironment remain to be established directly. These findings provide a basis for proposing that metabolically reprogrammed myeloid cells may influence B-cell function in GBM. We present these interactions as biologically plausible and therapeutically interesting while emphasizing that they require dedicated validation in GBM.

## 4 Macrophage–B cell crosstalk in GBM

### 4.1 Metabolic reprogramming and immunosuppressive signaling

Within GBM, TAMs have historically been described as predominantly m2-like, reflecting their frequently observed immunosuppressive and tumor supportive functions. However, it should be noted that the M1/M2 framework substantially oversimplifies TAM biology: single-cell and spatial studies of glioma consistently show that individual TAMs co-express canonical M1 and M2 programs and are more accurately described by ontogeny- and niche-defined states—including hypoxic, interferon-responsive, and lipid-laden populations—rather than by discrete polarization categories (48, 53, 54).

In the GBM microenvironment, both tumor-derived signals and immune cell interactions regulate TAM localization and function. B cells can influence macrophage recruitment and function through chemokines, antibodies, and other soluble mediators, while macrophages can reciprocally influence B-cell responses through soluble factors and antigen-presenting signals (55).

Metabolic and microenvironmental conditions further shape TAM behavior. Although GBM is generally associated with an immunosuppressive microenvironment, environmental factors such as oxygen availability and metabolic stress influence immune cell recruitment and function, including TAM localization and activity (55).

### 4.2 Nutrient competition and metabolic constraints

Tumor cells rely heavily on glycolysis and glutaminolysis, consuming large amounts of glucose and glutamine while producing lactate (56). This creates a hypoxic, nutrient-deficient TME that limits the availability of resources for immune cells (57). Activated T cells normally depend on glycolysis (after shifting from OXPHOS) to support proliferation and anti-tumor functions, but nutrient competition with tumor cells impairs their activity. GBM cells exhibit high glucose and glutamine utilization, and metabolic competition for these substrates contributes to immune disfunction within the GBM TME (58, 59). In contrast, TAMs can adapt by utilizing tumor-derived metabolites such as lactate and fatty acids. These metabolic conditions promote a shift toward an immunosuppressive and tumor-supporting phenotype, suppressing anti-tumor immunity and facilitating tumor progression (60).

B cells may also participate in this metabolic competition, although their specific metabolic responses within GBM remain poorly characterized. B-cell activation and differentiation require substantial metabolic reprogramming to support proliferation and effector functions, with nutrient requirements varying across B-cell states (61). Thus, the nutrient-depleted environment created by GBM may impose direct metabolic constraints on B cells in addition to the well-characterized effects on T cells and myeloid populations. Beyond glucose and arginine, two further metabolic axes contribute prominently to immunosuppression in GBM. First, tryptophan catabolism through the kynurenine pathway—initiated in tumor and myeloid cells by the enzymes IDO1 and TDO2—simultaneously depletes local tryptophan and generates kynurenine, an endogenous ligand of the aryl hydrocarbon receptor (AhR) (62). Tryptophan starvation and AhR engagement together drive CD8^+^ T-cell exhaustion and regulatory T-cell differentiation and reinforce immunosuppressive myeloid programs; in glioma, IDO1 and TDO expression rises with tumor grade and correlates with reduced survival (63, 64). Second, extracellular ATP released under hypoxia and cellular stress is sequentially hydrolyzed by the ectonucleotidases CD39 and CD73 to adenosine, which signals through the A2A receptor to raise intracellular cAMP, blunting effector T-cell and NK-cell responses while favoring regulatory T-cell and immunosuppressive macrophage phenotypes. CD73 is hypoxia-induced and highly expressed in GBM, and the CD39-CD73-A2AR axis is among the most consistently represented immunosuppressive programs across glioma-infiltrating immune cells (65). Because these pathways act on the same effector and regulatory populations that macrophages engage, they likely compound the arginine- and lactate-mediated constraints on lymphocyte function, although their specific impact on intratumoral B cells in GBM remains largely uncharacterized.

Because B cells function through antigen presentation, cytokine secretion, and interactions with T cells, metabolic constraints within the TME also indirectly influence B cell-mediated immune responses by limiting effective T-cell activation and effector function (66). However, B cells themselves undergo metabolic reprogramming upon activation and differentiation, increasing their metabolic demands to support proliferation and effector functions (61). Thus, B cells may also directly compete with tumor cells for nutrients withing the metabolically restrictive TMR, although the specific metabolic requirements of intratumoral B cells populations in GBM remain understudied. T-cell activation and differentiation are regulated by metabolic signaling pathways. Effector T-cell subsets depend heavily on glycolytic metabolism to support cytokine production and immune responses, whereas regulatory T cells rely more on oxidative metabolism and fatty acid oxidation. The activation and differentiation of these T cell populations rely on nutrient availability and cellular energy. Nutrient-poor conditions within the TME can impair effector T cell responses while favoring the persistence of immunosuppressive T cell populations. This shift in immune cell function contributes to tumor progression (67, 68). These metabolic constraints may therefore influence B cell mediated antitumor immunity both directly, through altered nutrient availability, and indirectly, through impaired T cells function. However, the direct effects of these metabolic constraints on B cells withing GBM have not been fully established (66).

### 4.3 Impaired B cell effector and antigen-presenting functions

In response to tumor-derived antigens, B cells undergo activation, clonal expansion, and differentiation into plasma cells and memory B cells. B cell activation is initiated through antigen recognition by the B cell receptor, which determines antigen specificity and triggers signaling pathways that promote proliferation and functional differentiation. During this process, activated B cells can migrate into secondary lymphoid organs or tertiary lymphoid structures, where they interact with other immune cells to coordinate adaptive immune responses (69). In addition to suppressing T cell responses, immunosuppressive myeloid populations such as monocytic MDSCs (M-MDSCs) can directly inhibit B cell proliferation and antibody production through the release of soluble suppressive mediators. These findings demonstrate that dysfunctional myeloid cells not only impair cellular immunity but also directly limit humoral immune responses, further weakening the ability of B cells to support macrophage-mediated tumor removal and adaptive immunity (70). Plasma cells produce tumor-specific antibodies that contribute to antitumor immunity through complement activation, antibody-dependent cellular cytotoxicity, and the opsonization of tumor cells (71).

Antibody-opsonized tumor cells are recognized by Fcγ receptors expressed on macrophages, promoting antibody-dependent cellular phagocytosis and enhancing tumor cell clearance (72–74). This antibody-mediated interaction between B cells and macrophages strengthens innate immune effector functions by facilitating the recognition and elimination of antibody-coated tumor cells (69).

Beyond antibody production, B cells also function as professional antigen-presenting cells, processing and presenting tumor antigens through MHC-II molecules to CD4^+^ T cells and supporting the generation of effective adaptive immune responses (69, 75). Unlike most lymphocytes, B cells possess the machinery required for antigen uptake and presentation, enabling them to directly activate CD4+ T cells and promote their proliferation and differentiation. Through MHC II-mediated antigen presentation, B cells coordinate with macrophages and other APCs to sustain adaptive antitumor immune responses (69). Collectively, impaired B-cell antibody production and antigen presentation can reduce macrophage-mediated phagocytosis, diminish T-cell activation, and weaken overall antitumor immunity, thereby facilitating tumor progression (76, 77).

As GBM progresses, metabolically reprogrammed tumor cells and TAMs contribute to the accumulation of lactate within the TME, creating an acidic, nutrient deprived environment that disrupts immune cell function. TAMs recognize acidic lactate through the surface receptor GPR132. This triggers an intracellular signaling cascade that stabilizes the transcription factor HIF-1α, completely independent of oxygen availability. HIF-1α binds to the promoter region of the Arg1 gene, inducing transcription. Importantly, this GPR132—HIF-1α—ARG1 pathway has been demonstrated primarily in breast cancer models, and its role in GBM remains to be established (78). TAMs then consume extracellular L-arginine, converting it into urea and L-ornithine, depleting extracellular arginine available to neighboring immune cells (79). Because activated B cells require extracellular arginine to support the metabolic demands of proliferation and differentiation, B cells require a rapid influx of extracellular arginine to initiate activation upon encountering an antigen. When TAMs deplete this amino acid pool, this may create a metabolic constraint on B-cell function (80). In the GBM microenvironment, this metabolic stress could potentially impair B-cell activation, proliferation, antibody production, and antigen-presenting capacity, reducing their ability to support macrophage-mediated phagocytosis and T-cell activation (81). Given the accumulation of lactate and other metabolic stressors within the GBM microenvironment, this pathway could potentially contribute to B-cell dysfunction in GBM; however, direct evidence for this mechanism in GBM remains limited. Such metabolic stressors may also potentially disrupt macrophage-B cell crosstalk and reinforce immune suppression (68, 69, 82).

### 4.4 Induction of regulatory B cell responses

Regulatory T cells, MDSCs, and TAMs can promote the development of Bregs through immunosuppressive signaling. As GBM progresses, TAMs increasingly adopt an immunosuppressive phenotype characterized by the production of anti-inflammatory mediators and reduced capacity to support effective immune responses. These changes establish a TME that favors immune tolerance and facilitates the differentiation of suppressive immune cell populations, including Bregs (83). These Bregs suppress anti-tumor T cell responses (82). No specific markers have been found to uniquely identify Bregs, making them difficult to study alone. They are identified by their typical characteristics, like their ability to produce suppressive molecules that reduce the activity of immune cells that would normally fight off tumors (82). In addition to limiting effector immune responses, TAM derived IL-10 and immune checkpoint molecules such as PD-L1 contribute to T cell dysfunction, reinforcing the immunosuppressive conditions that support regulatory immune populations (83).

Bregs can reinforce an immunosuppressive TME by promoting the expansion and activity of other regulatory immune cells. Tumors and TAMs, including MDSCs, can drive the development of Bregs through direct cell interactions and the release of signaling molecules (82). Beyond promoting regulatory B cell differentiation, MDSCs can also suppress conventional B cell effector functions. Experimental studies have shown that M-MDSCs inhibit B cell proliferation and antibody production, limiting effective humoral immunity through immunosuppressive mediators. Among these mediators, prostaglandin E_2_ (PGE_2_), produced by activated myeloid cells, suppresses B cell proliferation through signaling via the EP2 and EP4 receptors. These effects have been demonstrated in other tumor and disease models, and whether PGE2 mediates similar macrophage or MDSC-driven B-cell suppression remains unclear Because PGE2 is an immunosuppressive lipid mediator produced by myeloid cells, this pathway may represent an additional mechanism through which metabolically reprogrammed TAMs and MDSCs impair B cell responses within the GBM microenvironment (70). The acquisition and maintenance of this immunosuppressive TAM phenotype is regulated by intracellular signaling pathways that promote anti-inflammatory macrophage polarization and sustain the production of immunosuppressive mediators (83). In GBM, MDSCs have been shown to transfer suppressive signals to B cells, inducing a regulatory phenotype that further limits anti-tumor immune responses (82). Furthermore, IL-10 suppresses IL-12 production, reducing cytotoxic CD8+ T cell activity and further weakening antitumor immunity (83). These interactions create a cycle in which myeloid cells promote Breg development, while Bregs suppress cytotoxic immune cells and support tumor survival and progression (82). Collectively, these reciprocal interactions establish a self-sustaining immunosuppressive network that enables GBM cells to evade immune surveillance and promote continued tumor progression.

## 5 Perspective

GBM establishes a profoundly hostile TME characterized by hypoxia, nutrient deprivation, acidosis, and the accumulation of immunosuppressive metabolites such as lactate, kynurenine, and extracellular adenosine. While these metabolic conditions support tumor proliferation and invasion, they also profoundly reprogram the phenotype and function of surrounding immune cells. Tissue-resident microglia, infiltrating monocyte-derived macrophages, T cells, and B cells each undergo metabolic adaptations that ultimately favor immune evasion rather than effective anti-tumor immunity. Rather than acting independently, these immune populations exist within an interconnected metabolic network in which alterations in one cell type influence the function of others.

Standard radiochemotherapy is also an important determinant of the immune landscape in GBM. Although immunosuppressive mechanisms are present at diagnosis, radiation and temozolomide can further compromise systemic immune competence, including reductions in circulation T cells, B cells, and natural killer cells (84, 85). These treatment-associated changes are particularly relevant to macrophage and B cell biology. In recurrent GBM following standard therapy, monocyte-derived macrophages are continuously recruited, with an increased macrophage-to-microglia ratio compared with newly diagnosed tumors (86). At the same time, treatment-associated reductions in B cells and other lymphocyte populations may alter the cellular environment in which macrophage—B cell interactions occur (85). Thus, the clinical relevance of macrophage immunometabolism and its potential therapeutic modulation much be considered within the combined effects of tumor-induced and treatment-induced systemic immune dysfunction.

Throughout this review, we highlight macrophages, particularly tissue-resident microglia, as central integrators of these metabolic signals. Exposure to persistent hypoxia, glucose deprivation, altered lipid availability, and tumor-derived metabolites drives macrophages toward immunosuppressive phenotypes characterized by impaired antigen presentation, increased expression of inhibitory immune checkpoints, altered cytokine production, and enhanced secretion of factors that promote angiogenesis, extracellular matrix remodeling, and tumor invasion. These metabolically reprogrammed macrophages not only lose their capacity to initiate effective anti-tumor immune responses but also actively shape the behavior of adaptive immune cells. Through cytokines, chemokines, metabolic mediators, and direct cell-cell interactions, macrophages regulate B cell recruitment, activation, differentiation, and antibody production while simultaneously suppressing cytotoxic T-cell activation and persistence. Conversely, emerging evidence suggests that B cells can further influence macrophage polarization and function, highlighting the bidirectional nature of macrophage–B cell crosstalk within the GBM microenvironment.

Increasing evidence therefore suggests that macrophage immunometabolism represents a central mechanism linking innate and adaptive immune dysfunction in GBM. Rather than viewing macrophages solely as immunosuppressive effector cells, they should be considered metabolic orchestrators that translate environmental stress into widespread immune remodeling. Defining the molecular pathways that govern these interactions—including HIF signaling, lipid metabolism, amino acid utilization, mitochondrial function, and immunoregulatory metabolite production—may reveal opportunities to simultaneously restore macrophage inflammatory activity, enhance B-cell-mediated antigen presentation and humoral immunity, and improve T-cell effector function.

Future therapeutic strategies will likely require targeting these interconnected metabolic networks rather than individual immune populations in isolation. Combining metabolic reprogramming of macrophages with immunotherapies that enhance B cell and T-cell responses may overcome the profound immune suppression that has limited the success of current GBM treatments. As our understanding of tissue-resident macrophage metabolism and macrophage–B cell communication continues to evolve, these interactions represent a promising avenue for developing more effective immunometabolic therapies capable of reshaping the GBM immune landscape and improving patient outcomes.

### 5.1 Concluding remarks

The growing understanding of macrophage immunometabolism has shifted the view of microglia from passive components of the GBM microenvironment to active regulators of tumor progression and immune function. Rather than acting solely through inflammatory signaling, metabolic reprogramming influences how resident microglia and infiltrating macrophages communicate with surrounding immune cells, shaping the balance between immune activation and suppression. Although considerable progress has been made in defining the metabolic and functional diversity of TAMs, many aspects of their interactions with adaptive immune cells remain poorly understood. Further characterization of these pathways may identify new opportunities to selectively reprogram TAMs while preserving the essential homeostatic functions of resident microglia. A better understanding of these metabolic interactions will be important for the development of more effective immunotherapeutic strategies for GBM.
